# Integrated Analysis of Whole Genome and Transcriptome Sequencing Reveals Diverse Transcriptomic Aberrations Driven by Somatic Genomic Changes in Liver Cancers

**DOI:** 10.1371/journal.pone.0114263

**Published:** 2014-12-19

**Authors:** Yuichi Shiraishi, Akihiro Fujimoto, Mayuko Furuta, Hiroko Tanaka, Ken-ichi Chiba, Keith A. Boroevich, Tetsuo Abe, Yoshiiku Kawakami, Masaki Ueno, Kunihito Gotoh, Shun-ichi Ariizumi, Tetsuo Shibuya, Kaoru Nakano, Aya Sasaki, Kazuhiro Maejima, Rina Kitada, Shinya Hayami, Yoshinobu Shigekawa, Shigeru Marubashi, Terumasa Yamada, Michiaki Kubo, Osamu Ishikawa, Hiroshi Aikata, Koji Arihiro, Hideki Ohdan, Masakazu Yamamoto, Hiroki Yamaue, Kazuaki Chayama, Tatsuhiko Tsunoda, Satoru Miyano, Hidewaki Nakagawa

**Affiliations:** 1 Laboratory of DNA Information Analysis, Human Genome Center, The Institute of Medical Science, The University of Tokyo, Tokyo, 108-8639, Japan; 2 Laboratory for Genome Sequencing Analysis, RIEKN Center for Integrative Medical Sciences, Yokohama, 230-0045, Japan; 3 Laboratory for Medical Science Mathematics, RIEKN Center for Integrative Medical Sciences, Yokohama, 230-0045, Japan; 4 Laboratory of Sequence Analysis, Human Genome Center, The Institute of Medical Science, The University of Tokyo, Tokyo, 108-8639, Japan; 5 Department of Medicine & Molecular Science, Hiroshima University School of Medicine, Hiroshima, 734-8551, Japan; 6 Second Department of Surgery, Wakayama Medical University, Wakayama, 641-8510, Japan; 7 Department of Surgery, Osaka Medical Center for Cancer and Cardiovascular Diseases, Osaka, 537-8511, Japan; 8 Department of Surgery, Institute of Gastroenterology, Tokyo Women's Medical University, Tokyo, 162-8666, Japan; 9 Department of Anatomical Pathology, Hiroshima University School of Medicine, Hiroshima, 734-8551, Japan; 10 Department of Gastroenterological Surgery, Hiroshima University School of Medicine, Hiroshima, 734-8551, Japan; 11 Laboratory for Genotyping Development, RIEKN Center for Integrative Medical Sciences, Yokohama, 230-0045, Japan; Baylor College of Medicine, United States of America

## Abstract

Recent studies applying high-throughput sequencing technologies have identified several recurrently mutated genes and pathways in multiple cancer genomes. However, transcriptional consequences from these genomic alterations in cancer genome remain unclear. In this study, we performed integrated and comparative analyses of whole genomes and transcriptomes of 22 hepatitis B virus (HBV)-related hepatocellular carcinomas (HCCs) and their matched controls. Comparison of whole genome sequence (WGS) and RNA-Seq revealed much evidence that various types of genomic mutations triggered diverse transcriptional changes. Not only splice-site mutations, but also silent mutations in coding regions, deep intronic mutations and structural changes caused splicing aberrations. HBV integrations generated diverse patterns of virus-human fusion transcripts depending on affected gene, such as *TERT*, *CDK15*, *FN1* and *MLL4*. Structural variations could drive over-expression of genes such as WNT ligands, with/without creating gene fusions. Furthermore, by taking account of genomic mutations causing transcriptional aberrations, we could improve the sensitivity of deleterious mutation detection in known cancer driver genes (*TP53, AXIN1, ARID2, RPS6KA3*), and identified recurrent disruptions in putative cancer driver genes such as *HNF4A*, *CPS1*, *TSC1* and *THRAP3* in HCCs. These findings indicate genomic alterations in cancer genome have diverse transcriptomic effects, and integrated analysis of WGS and RNA-Seq can facilitate the interpretation of a large number of genomic alterations detected in cancer genome.

## Introduction

Each year, more than half a million people worldwide are diagnosed with hepatocellular carcinoma (HCC), the fifth and seventh most common cancer in men and women, respectively [1]. In most cases, HCCs develop following hepatitis or cirrhosis caused by hepatitis B virus (HBV) infection, hepatitis C virus infection, alcoholism, or metabolic diseases, of which HBV is the most major factor, especially in South-East Asia and sub-Saharan Africa [1]. Although various genetic alternations have been detected in HCCs, such as mutations of *TP53* and *CTNNB1* encoding β-catenin [2], further detailed characterization of liver cancer genome is required for identification of biomarkers for personalized medicine and more effective therapeutic drug development.

Recent advances in high-throughput sequencing technologies enable us comprehensive detection of somatic mutations in cancer genomes [3] and the high-throughput sequencing of HCC genomes has revealed several novel cancer driver genes such as chromatin regulators [4], [5] and recurrent virus integrations at the *TERT* and *MLL4* loci [4], [6]–[8]. Current genomic studies mainly focus on mutations in coding regions, and other types of mutations such as base substitutions or indels in non-coding regions, and structural variations (SVs) are usually ignored, since their impact on cancer development is difficult to evaluate and interpret so far. One approach for evaluating the deleteriousness of these mutations is to check the transcriptional consequences of these genomic alterations. For this purpose, broader understandings of the relationships between genomic mutations and transcriptional aberrations in cancer genome are necessary. Several examples of splicing aberrations [9], [10] and gene fusions [11] caused by genomic mutations are known, and studies using recent high-throughput sequencing data identified cancer-specific transcriptional aberrations in several cancer types [12], [13]. However, there are still few studies that systematically compare genomic mutations and transcriptional aberrations from whole genome sequencing (WGS) and transcriptome sequencing (RNA-Seq) data. As such, we still have little knowledge on the landscape of the cancer transcriptome and its relationships with somatic mutations.

**Figure 1:**
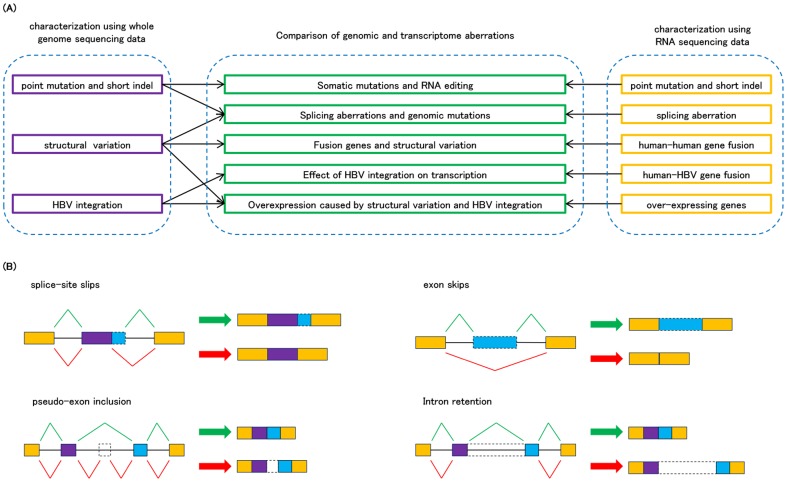
The outline of theRNA-Seq study integrated with whole genome sequencing. (A) First, we detected various types of genomic and transcriptomic changes from RNA-Seq data of 22 HCCs. The characterized changes detected by each analysis were compared to reveal the effects of somatic genomic changes on transcriptomic aberrations. (B) The four types of splicing aberrations defined in this study. Green lines and arrows indicate normal transcription whereas red lines and arrows indicate aberrant transcriptions.

Previously, we sequenced and analyzed WGS of 27 diverse types of liver cancers [4], but the effects of large part of diverse somatic mutations, including non-coding mutations and SVs, were hardly to interpret only using the WGS data. Therefore, in this study, we added more WGS and their corresponding RNA-Seq data, totally from 22 HBV-related HCC samples, to determine the genetic alterations together with their transcriptional consequence. and we performed comparative and integrated analyses of their WGS and RNA-Seq data (see **Fig. 1A** for the overview of the study). First, we identified a variety of somatic genomic events including point mutations, short indels, SVs, and HBV integrations from WGS data. Then, after systematically characterizing cancer-specific transcriptomic aberrations, such as various types of splicing alterations (exon skips, splice-site slips, pseudo-exon inclusions and intron retentions, see **Fig. 1B**), gene fusions including those involving HBV sequences, over-expression events and nucleotide changes at RNA level, we investigated relationships between detected genomic and transcriptomic changes. Finally, providing a profile of genomic mutations and transcriptional aberrations, we discuss the benefits of integrated analysis of WGS and WTS for sensitive detection of cancer driver genes.

## Results

### Somatic events detected by WGS

We extracted DNA from 22 frozen HBV-related HCC tissues and their matched normal lymphocytes, and sequenced their whole genomes by massive parallel sequencing. Clinical and pathological information is shown in **Table S1 in S1 File**. Average sequencing depths of the cancer and control (lymphocytes) genomes were 36.8× and 29.9x, respectively, after the removal of PCR duplications (**Table S2 in S1 File**). In total, 209,055 (3,597–19,063) somatic substitutions were detected (**Table S4 in S1 File**). Among them, 1,096 missense, 32 nonsense and 35 splice-site mutations in protein-coding regions, were identified, where splice-site mutations were defined to be those affecting splice acceptor and donor sites located at the first and last two bases of an intron sequence (essential splice-sites). Of the 5,725 indels identified in the whole genomes, 105 were located in protein-coding regions and 6 affected essential splice-sites. The inferred driver genes in order of statistical significance of recurrence were *TP53*, *ARID2*, *BRD7*, *HNF4A* and *RPS6KA3* (*P*-value <0.001, **Table S5 in S1 File**). In addition, 2,254 SVs (15–577 per tumor) were detected, 1,168 of which affected annotated protein-coding genes. Furthermore, 86 (0–12 per tumor) HBV integrations were identified with 2 and 5 recurrent integrations at the *TERT* and *MLL4* loci, respectively, which is consistent with previous studies [4], [6], [7], [8].

### Splicing aberrations related with genomic mutations

Total RNAs extracted from the frozen 22 HCCs and their adjacent non-cancerous liver tissues were subject to RNA-Seq, and its summary is shown in **Table S3 in S1 File**.

First, we investigated the status of transcripts around essential splice-site mutations by manually checking the alignments of sequence reads. After excluding transcripts with no expression, we observed single or multiple splicing aberrations for 19 out of the remaining 24 essential splice-site mutations (3 splice-site slips, 6 exon skips, and 13 intron retentions, **Figure S1 in S2 File**, **Table S6 in S1 File**), indicating that the somatic mutations at essential splice-sites showed strong effects on splicing aberrations as expected. Affected genes included recurrently mutated genes from WGS analysis (*TP53, ARID2, HNF4A* and *RPS6KA3*) as well as *AXIN1*.

In order to obtain a comprehensive list of cancer-specific splicing aberrations, we systematically detected four types of splicing aberrations (splice-site slip, exon skip, pseudo-exon inclusion and intron retention, **Fig. 1B**) by using internally developed algorithms (see **Materials and Methods**). Overall, 292 splicing aberrations events (26 splice-site slips, 41 exon skips, 77 pseudo-exon inclusions and 148 intron retentions) were detected (**Table S7 in S1 File**). Since we performed non-directional RNA-Seq, discriminating between intron retention and cancer-specific antisense transcription was difficult, and thus the results of intron retentions should be carefully interpreted. This list included aberration events corresponding to the 10 essential splice-sites investigated above. PCR and Sanger sequencing validated 154 highly cancer-specific splicing events out of the 239 detected. For 72 events, we confirmed target splicing aberrations for both cancer and non-cancerous liver tissues.

**Figure 2:**
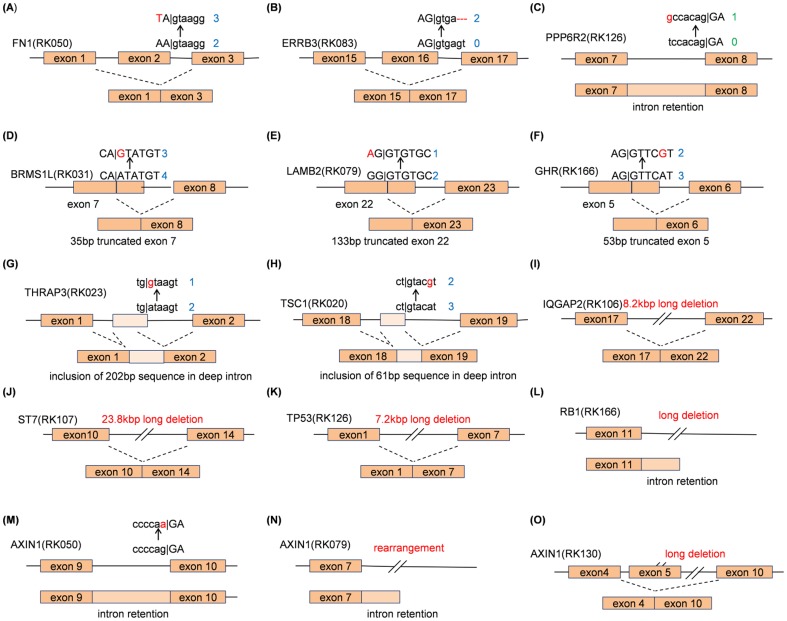
Several examples of genomic changes other than essential splice-site mutations causing splicing aberrations obtained from our comparative whole genome and transcriptome sequencing analyses. Exonic and intronic sequences are designated by capital and small letters, respectively. Red sequences are somatic mutations in HCCs. Blue and green numbers on the side of sequences are edit distances from splicing donor motif (AG|GTRAGT, [38]) and splicing acceptor motif (YYYYNCAG|G), respectively. Most somatic mutations changed the edit distance to splicing donor motifs so that the corresponding alteration can be enhanced.

In addition to essential splice-site mutations, we identified various types of mutations and short indels that appear to be the direct causes of the observed splicing aberrations, through changing the edit distances of splicing donor or acceptor motifs. Three mutations near exon-intron junctions (<10 bp), but not in the essential splice-site caused splicing aberrations (**Fig. 2A, B, and C**). Three mutations in coding regions, which likely generated novel splice-site donor motifs, caused splice-site slips (**Fig. 2D, E, and F** **)**. One mutation affecting the *LAMB2* gene was synonymous. Two pseudo-exon inclusion event affecting *THRAP3* and *TSC1* seemed to be triggered by somatic mutations deep within introns proximal to the new splicing junction point (**Fig. 2G, and H**). Furthermore, seven exon skips, whose affected genes included several tumor suppressor genes such as *IQGAP2*, *ST7* and *TP53*, had long deletions between the junction points (**Fig. 2I, J, and K**). In addition, eight intron retentions had rearrangements within the corresponding introns, for example *RB1* (**Fig. 2L**). They indicate that various types of splicing aberrations are frequently driven by not only essential splice-site mutations, but also mutations in coding regions, including synonymous silent mutations, deep intronic mutations, and SVs. On the other hand, a large part of splicing aberrations (239/292 = 81.8%) did not have proximal mutations (within 1 kb) or SVs (within 500 kb). Some of these are likely seemed to be caused by epigenetic changes [14], or expressional changes in anti-sense transcripts as noted above.

### Fusion transcripts related with genomic SVs

To detect gene fusions with unannotated transcripts and/or viral sequences such as HBV, we used Genomon-fusion (see **Materials and Methods**). We detected 245 candidates of human-human fusion transcripts and 192 gene fusions after removing redundant splicing variants (**Figure S2 in S2 File** and **Table S8 in S1 File**), 66 of which involved transcripts without gene annotation (UCSC known genes, RefSeq, Ensemble), and 21 of which were un-spliced fusion transcripts sharing the breakpoints with their corresponding genomic SVs (**Figure S3 in S2 File**). RT-PCR followed by Sanger sequencing validated 113 (71.9%) of 157 fusion transcripts.

Through comparison with WGS data, 83 gene fusions were found to be supported by somatic SVs at the corresponding genomic locations (**Figure S4 in S2 File**). While some of gene fusions without observed corresponding SVs may be ascribed to either false positives for gene fusions in RNA-Seq analysis or false negatives for SVs in WGS analysis, ratios of expression values of fusion transcripts imply the existence of minor sub-clones with undetectable associated SVs (**Figure S5 in S2 File**). We also detected 147 gene fusions in non-tumor liver tissues (**Table S9 in S1 File**), many of which involved genes with extremely high expression values in liver tissues, such as *ALB*, *HP*, and *TF*, suggesting that detected fusion transcripts may also have originated from SVs harbored within minor sub-clonal liver cells (**Figure S5 in S2 File**).

**Figure 3:**
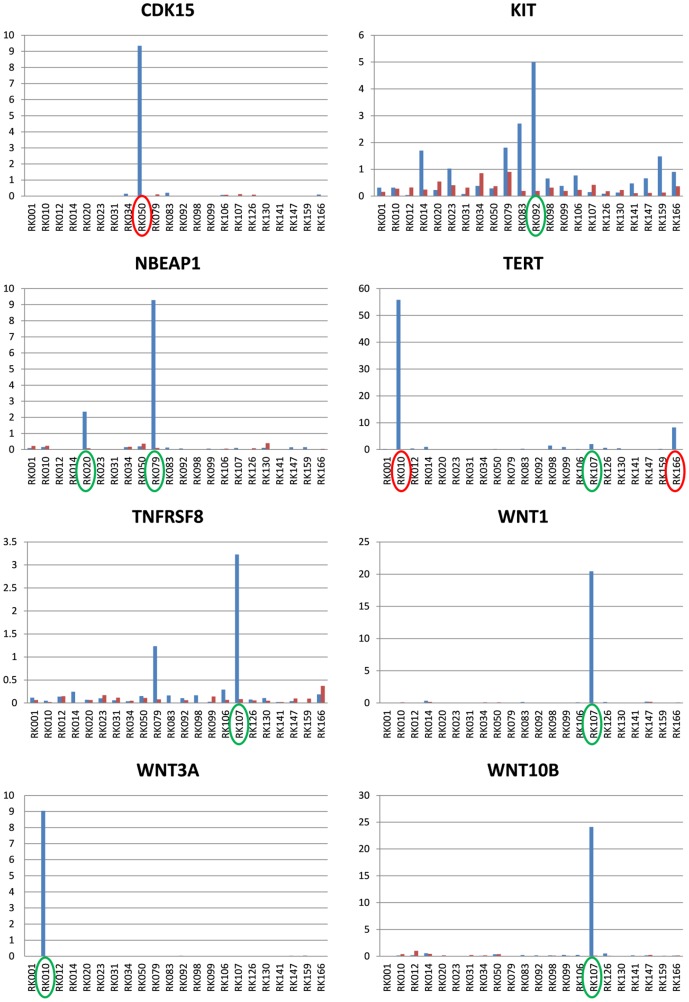
The expression profiles of 22 HCCs and non-cancerous liver samples for eight over-expressed genes. Blue and red bars show the FKPMs for HCCs and the corresponding non-cancerous liver, respectively, which is calculated by RNA-Seq data. Red circles indicate samples with HBV integrations on the loci of the overexpressed genes. Green circles indicate those with gene fusions and/or SVs that can drive gene over-expression.

Among them, *NBEAP1* (*BCL-8*) fusion transcripts were recurrently detected and validated in two HCCs, with over-expression specific to both specimens (**Fig. 3** and **Figure S6 in S2 File**). Rearrangements involving the *BCL-8* locus with over-expression were reported to occur in about 4% of diffuse large-cell lymphoma [15]. Many fusion transcripts affecting chromatin modification pathway genes (*CHD4*, *CTCF*, *KDM4C* and *HDAC4*) were detected, and fusion transcripts with known tumor suppressor genes (*TSC1* and *SUFU*), a component of the crucial NF-κB modulator (*IKBKB*), and a key meditator of the WNT signaling pathway (*TCF7L1*) were also validated [16]. Although no specific over-expression resulted from these gene fusions, we speculate several of them have a loss-of-function nature though the loss of physiologically important domains (**Figure S7 in S2 File**).

### HBV integration and its effects on transcription

Overall, 33 HBV-human fusions were detected, including those affecting *TERT* (2 samples) and *MLL4* (5 samples), and WGS could identify associated HBV integration sites for 23 of these 33 fusions (**Table S10 in S1 File**). HBV integrations with associated gene fusions tended to have breakpoints concentrated around the locus of HBx genes (1770 bp–1830 bp) in the positive direction (**Figure S8 in S2 File**), as reported previously [8].

**Figure 4:**
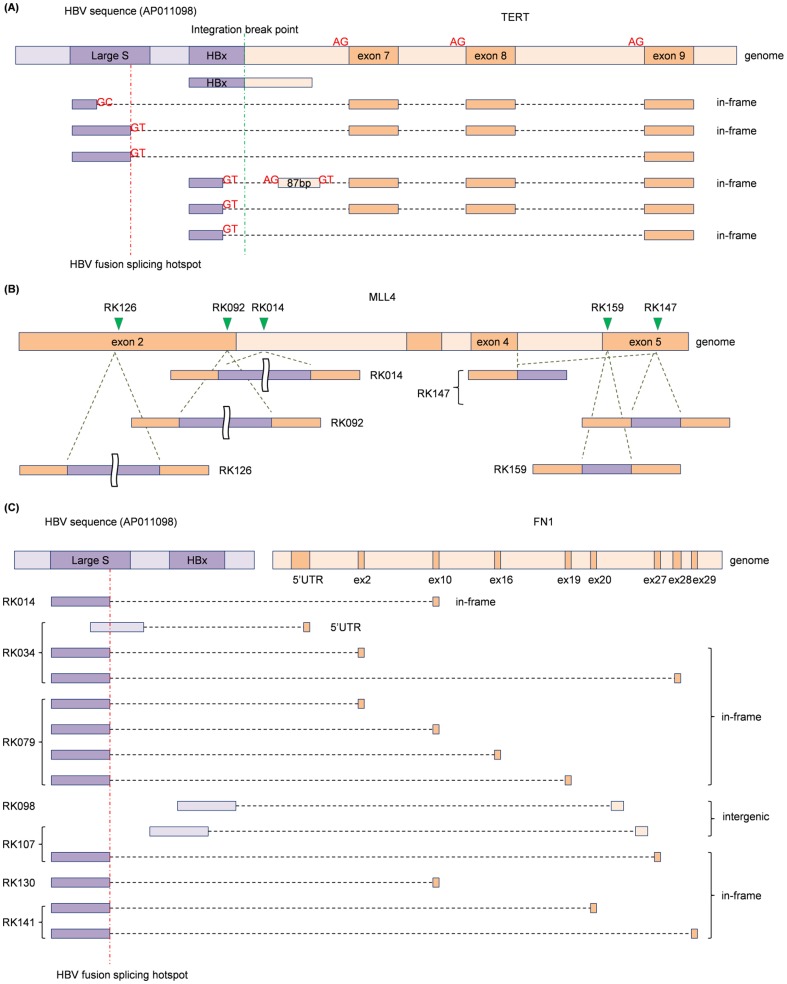
HBV integrations and fusion events in 22 HCCs. (A) Seven *HBV-TERT* fusion transcripts were detected in RK010. One transcript was an un-spliced transcript having the same breakpoint as the genomic integration breakpoint. The others existed in spliced forms and GT-AG splicing motifs were observed at the breakpoints of all but one. In addition to HBV fusion splicing hotspot (458 bp), 3 fusion transcripts were spliced at the coordinate of 1634 bp coordinates in HBV sequences. One fusion transcript included a newly generated 87 bp pseudo-exon sequence as well as subsequence exonic sequences. (B) HBV integrations in the *MLL4* loci and their resultant fusion transcripts in five samples. Green triangles on the genome sequence show the HBV integration sites. Most fusion transcripts shared breakpoints with those of genomic HBV integration coordinates for both sides, and thus, they appear to exist in un-spliced forms. The fusion transcripts for RK141 and RK159 were validated to be concatenated (**Figure S11**). (C) *HBV-FN1* fusion transcripts for 7 adjacent non-cancerous liver samples. Almost all the fusion transcripts had the breakpoint at the HBV fusion splicing hotspot. The other fusion transcripts which had breakpoints at intronic regions appear to be un-spliced transcripts around the integration sites.

Interestingly, 7 discrete HBV*-TERT* fusion transcripts were detected in one sample (RK010), which appeared to be derived from one HBV integration site (**Fig. 4A**). 4 out of the 7 variants are inferred to be in-frame. RK166 had HBV integration just before the transcription start sites of *TERT*, which generated the full *TERT* transcript directly connected to HBV sequence (**Figure S9 in S2 File**). Both samples showed a marked over-expression of *TERT* compared to other samples (**Fig. 3**). A HBV*-CDK15* gene fusion detected in RK050 also had multiple fusion transcripts including one in-frame fusion which caused *CDK15* over-expression (**Figure S10 in S2 File**). The breakpoints of spliced HBV-human fusion transcripts were concentrated at the HBV genome coordinate of 458bp. We call this position the HBV fusion splicing hotspot.

On the other hand, gene fusions involving *MLL4* showed different patterns. While most observed gene fusion contained HBV on the 5′ end, we detected two types of HBV-*MLL4* gene fusions: those with HBV on the 5′ side and those with HBV on the 3′ side (**Fig. 4B**). PCR with subsequent Sanger sequencing validated that these were parts of concatenated unspliced fusion transcripts of *MLL4-*HBV*-MLL4* for at least two samples (**Figure S11 in S2 File**). No evidence of splicing was obtained for these fusion transcripts except for one in RK141. Although slightly increased expression of *MLL4* was observed in HCC samples, these out-of-frame HBV*-MLL4* fusion transcripts suggest that HBV integrations on *MLL4* loci may lead to loss-of-function.

In non-cancerous liver tissues, 161 HBV-human fusion transcripts were detected (**Figure S12 in** **S2 File** and **Table S11 in** **S1 File**). Notably, HBV-*FN1* gene fusions were recurrently observed in 7 non-cancerous liver tissues, and most of them had multiple splicing variants including in-frame fusion transcripts with the HBV fusion splicing hotspot as in the HBV-*TERT* fusion (**Fig. 4C**).

### Over-expression caused by somatic SVs

There are several documented examples of chromosomal SVs leading to ectopic expression of downstream oncogenes with and without the formation of gene fusions [11]. Here, we examined the relationships between somatic SVs and over-expression of genes adjacent to the breakpoints. Through iterative application of the Grubbs-Smirnov test, we identified 3,735 over-expression events in 3017 genes in 22 HBV-related HCCs. The list of over-expression events included *TERT* and *CDK15* over-expression driven by HBV integration. Proximal SVs and HBV integrations (within gene regions or 500 kb up-stream from transcription start sites) could be associated with 63 and 5 over-expression events, respectively (**Table S12 in S1 File**). Of those breakpoints, 44 events occurred within the promoter regions, suggesting mechanisms of over-expression other than gene fusions are common phenomena. Although it is difficult to confirm each observed over-expression event is truly the consequence of the identified SVs, statistical significance by permutation test (*P*-value <0.0001, **Figure S13 in S2 File**) indicates many over-expressing events could be driven by somatic SVs.

Interestingly, over-expression events of WNT ligands were recurrently observed in two HCCs. Over-expression of *WNT1* and *WNT10B* (**Fig. 3**) in RK107 had associated SVs. Although a gene fusion involving *WNT10B* was observed, this did not seem to be the direct cause of over-expression because *WNT10B* was the upstream side of fusion transcripts (**Figure S14 in S2 File**). For *WNT3A* overexpression in RK010 (**Fig. 3**), we detected fusion transcripts involving *WNT3A* and a few supporting read pairs for them in WGS data, suggesting complex rearrangements around the *WNT3A* locus may drive its over-expression. Furthermore, the over-expression of *c-KIT* with associated SV could be detected in RK092 (**Fig. 3**). The above findings indicate that SVs can play an important role in over-expression of oncogenes and molecular target genes.

### Complementary detection of somatic mutations and cancer-specific RNA-editing events

We investigated cancer-specific SNVs (single nucleotide variant) and short indels in the RNA-Seq data using the EBCall algorithm [17], and detected 6,024 candidates at RNA levels, including 1,205 nonsynonymous or splice-site mutations. Among them, WGS analysis integrated with RNA-Seq data (See **Materials and Methods**) indicated evidences of 1,912 somatic mutations (545 nonsynonymous or splice-sites). There is a certain level correlation between the allele frequencies of somatic mutations found to be highly confident in WGS and RNA-Seq (correlation efficiency  = 0.466, *P-*value  = 2.2×10^−16^ by Pearson's product-moment correlation, **Figure S15 in S2 File**), but the amount of correlation is not very strong. This may be because changes of post-transcriptional processes such as non-sense medicated decay or mRNA stabilization brought by somatic mutations. Of these, 417 (112 nonsynonymous or splice-sites) were not called as somatic mutations in initial WGS analysis without considering RNA-Seq data, including several known driver genes such as *CTNNB1* and *TSC2*. Many of the somatic mutations detected by this integrative analysis were confirmed by Sanger sequencing of cancer DNAs (74/83 = 89.1%), and some of the unconfirmed mutations may be below the detection limit of Sanger sequencing owing to their low clonal proportion. Therefore, some of the false-negative somatic mutations resultant from the low sequencing coverage in WGS analysis can be rescued by complementary RNA-Seq analysis.

**Figure 5:**
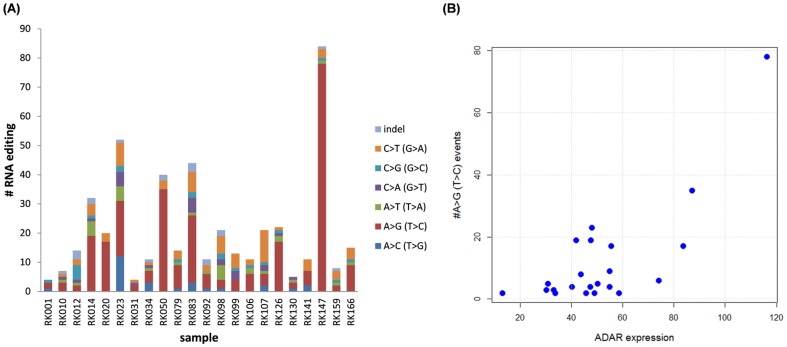
RNA editing candidates in 22 HCCs. (A) The number of cancer-specific RNA mutation events (RNA editing candidates) and their substitution patterns for each sample. (B) Scatter plot between the number of A:T>G:C RNA-editing events and *ADAR* expression value (FKPM) calculated by whole transcriptome sequence data. There is a significant correlation (*P*-value  = 2.38×10^−7^ by Wilcoxon rank sum test) between the number of A:T>G:C events and *ADAR* expression levels.

Finally, with no evidence of supporting variant reads in neither tumor nor normal WGS data, we identified 464 cancer RNA-specific events that are candidates for RNA-editing [18]. Although it is difficult to confirm the authenticity of each editing event, the mutation profile was abundant in A:T>G:C patterns and occurred in 3′ UTR regions, indicating that many of them are likely to be caused through RNA-editing by ADARs (adenosine deaminases) at the post-transcription stage [18] (**Fig. 5A**). The number of candidate RNA-editing events varied widely among the samples. We found a significant correlation (*P*-value = 2.38 ×10^−7^ by Wilcoxon rank sum test) between the number of A:T>G:C events and *ADAR* expression levels (**Fig. 5B**), implying the existence of new cancer subtypes determined by the amount of somatic RNA-editing.

### Landscape of genomic and transcriptomic disruptions in HBV-related HCCs

Considering the fact that many transcriptomic aberrations are caused by genomic changes, the detection of transcriptomic alterations, such as splicing aberrations and fusion transcripts, raises high probability of existence of proximal genomic changes. From this aspect, by complementary use of RNA-Seq to WGS analysis, we further rescued 64 combinations of genomic mutations and associated transcriptional aberrations (see **Materials and Methods**). In total, 252 genomic mutations causing transcriptional aberrations (GMTAs) were detected.

Through this integrated analysis of WGS and RNA-Seq, we could obtain comprehensive profiles of genomic and transcriptomic changes such as point mutations, indels, structural variations, splicing aberrations and gene fusions for each affected gene. Here, a critical problem is to discriminate cancer drivers from merely passenger events by using these profiles. In fact, although recurrent SVs (> = 3 HCCs) were seen in 12 genes (*C10orf11*, *CIT*, *CLTC*, *CNTNAP2*, *DSCAML1*, *EYS*, *FHIT*, *LARGE*, *LRP1B*, *MACROD2*, *MAGI3*, *TTC28*), many of them are located on or very proximal to common fragile regions [19] and actually were not found to have any influence on transcription (**Figure S16A in S2 File**), implying most of them are passenger SVs which occurred in unstable genomic regions. In order to remove passenger events, we only considered SVs evidenced by associated transcriptional aberrations, which is also helpful for removing false-positive detections in WGS analysis. On the other hand, recurrent gene fusions (> = 3 HCCs) were identified in 6 genes (*ALB, CES1, FGA, SEPP1, SERPINA1,* and *TF*). WGS analysis did not detected any SV associated with these fusions (**Figure S16B in S2 File**), implying that these fusions seem to come from minor sub-clonal cells or artifacts, and may not be driving forces for clonal expansion of cancer cells. These observations also support the importance of combinations of transcriptional aberrations and associated genomic mutations.

**Figure 6:**
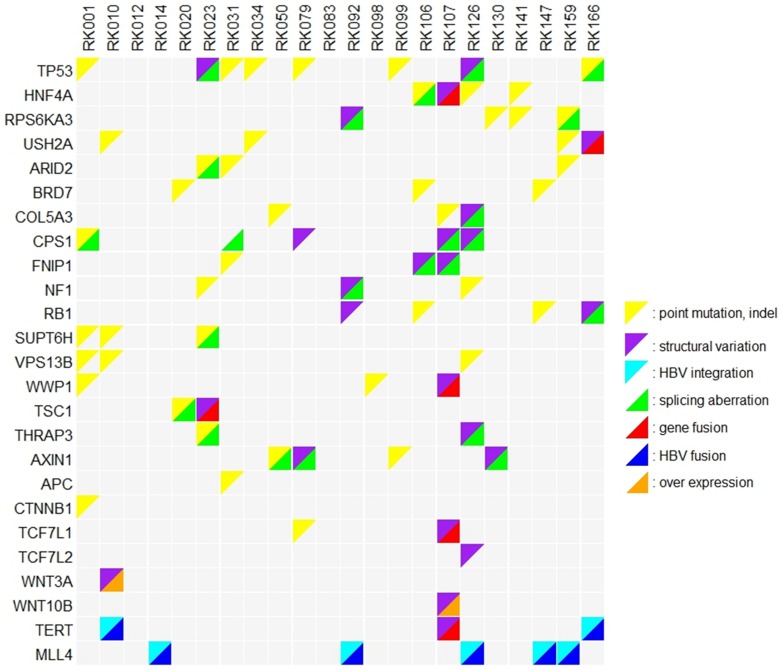
The status of genomic and transcriptomic alterations of representative genes, detected by WGS and RNA-Seq of 22 HBV-related HCCs. The list of genes were extracted by (1) significantly mutated genes in WGS analysis, (2) having no less than 3 mutations (point mutations or indels in coding regions, or GMTAs), (3) having no less than 2 GMTAs and registered in cancer gene census [17]. (4) involved in WNT signaling pathway, (5) *TERT* or *MLL4*.

In this WGS analysis we found that GMTAs were concentrated on significantly mutated genes (three in *TP53*, two in *HNF4A* and *RPS6KA3*, one in *ARID2*), indicating their implication in cancer pathogenesis (**Fig. 6**). Among the above eight GMTAs, four were SVs and could not be detected by sole investigation of coding regions, suggesting that combination of WGS and RNA-Seq analysis is effective to detect candidates driver genes. Thus, *HNF4A* is likely to be a novel driver gene for liver cancer, as well as *ARID2* [4] and *RPS6KA3* [20]. *HNF4A* plays a critical role in the regulation of multiple metabolic pathways in the liver as well as hepatocyte differentiation, and down-regulation of *HNF4A* has been shown to be associated with HCC [21], [22]. Furthermore, key genes in the WNT signaling pathway (*APC, AXIN1, CTNNB1, TCF7L1, TCF7L2* and WNT ligands) were frequently mutated (11 mutations) in nine HCCs, six of which affected their transcriptional consequences as GMTAs.

## Discussions

Through comparative and integrative analysis of WGS and RNA-Seq, we obtained a number of evidence that genomic mutations, including non-coding mutations, SVs and virus integrations, can cause diverse transcriptomic aberrations, such as splicing changes, gene fusions and over-expressions. In spite of much evidence that synonymous silent mutations in coding regions and deep intronic mutations lead serious diseases by disrupting transcription [23]–[25], they are often ignored in current cancer genome sequencing studies, and the same holds for SVs. Therefore, performing RNA-Seq combined with WGS is essential to interpret the consequences of somatic alterations including those in non-coding regions and SVs in cancer genomes. In addition, by using WGS and RNA-Seq complementary, we rescued not only a number of additional somatic mutations but also splicing aberrations caused by genomic mutations, that were narrowly missed the criteria for being called by single analysis.

In liver cancer genome, HBV integrations were frequently observed as one of SVs and in this study we observed that HBV integrations caused diverse transcrptic alterations such as virus-human fusions. Interestingly, we also observed recurrent HBV-*FN1* fusion events in non-cancer liver tissues. Although the previous studies also indicated that HBV integrations in the *FN1* loci could be specific to adjacent non-cancerous liver tissues at the genome level [6], [7], they did not detected resultant fusion transcripts. The frequency of HBV-*FN1* fusion in the present study (31.8% = 7/22) was much higher than those of previous studies (5.6% ( = 5/88) [6] and 10.0% ( = 4/40) [7]). This mainly seems to be because HBV integrations in the *FN1* locus are often harbored in minor sub-clonal populations in non-cancerous liver cells, which are often missed by the low-depth genome sequencing. However, RNA-Seq analysis allows for more sensitive prediction of sub-clonal HBV integration events when they generate a large enough amount of aberrant transcripts. Paradoxically, the fact that HBV integrations in the *FN1* loci does not occur in tumor samples implies that *FN1* is not simply prone to random HBV integrations; HBV-*FN1* fusion transcripts may play important roles in liver fibrosis or cirrhosis, or may enhance cancer development of cells proximate to those with HBV-*FN1* fusions.

In this study, we demonstrated that taking account of GMTAs, which can be effectively detected by comparative WGS and RNA-Seq analysis, leads sensitive detection of disruptions in cancer driver genes. Recurrent GMTAs were observed in genes other than those significantly mutated genes in WGS, including *CPS1*, *TSC1*, and *THRAP3*. *CPS1* is the key enzyme in the urea cycle, converting ammonium into carbamoyl phosphate. In HCCs, *CPS1* was reported to be down-regulated by DNA methylation [26]. We observed four mutations including three GMTAs in *CPS1*: splice-site mutation and long deletion causing exon skips, and translocation generating fusion transcripts for *CPS1*. *TSC1*, a key modulator of the mTOR pathway, has been implicated as a tumor suppressor in many types of cancers [27], and *TSC1* mutations in bladder cancers are shown to be related to everolimus sensitivity [28]. We identified two GMTAs in *TSC1*: one deep intronic mutation causing pseudo-exon inclusion and one translocation leading to gene fusion were observed. *THRAP3*, a member of the thyroid hormone receptor-associated protein (TRAP) complex, is implicated in pre-mRNA splicing, post-transcriptional mRNA degradation, and DNA damage response pathway [29], [30]. HBV integration at the *THRAP3* locus was also reported [31], as well as mutations in other cancers. We observed two GMTAs in *THRAP3*: one deep intronic mutation leading to pseudo-exon inclusion and one long deletion leading to exon skip. They are good candidates of driver genes for liver cancer.

In summary, our integrative and comparative analysis with WGS and RNA-Seq indicated genomic alterations in cancer genome have diverse transcriptomic effects, and this approach can improve detection of deleterious mutations and facilitate the interpretation of a large number of genomic alterations in cancer genome. There is still a room for algorithmic improvement in systematic and accurate detection of transcriptomic aberrations, and some classes of aberrations may not be detectable due to the limitations of short-read sequencing data. However, integrative analysis of WGS and RNA-Seq is a crucial approach for interpreting cancer genomes and understanding cancer biology underlying genomic alterations.

## Materials and Methods

### Clinical samples

The clinical and pathological features of the 22 HBV-related HCCs are shown in **Table S1 in File S1**. WGS analysis on RK001, RK010, RK023, and RK034 were reported in our previous study [4], Hepatitis B surface antigen (HBsAg) in the serum of all samples except for RK001. In RK001, HBV-DNA was detected by sensitive PCR in serum, indicating occult HBV infection. All subjects had undergone partial hepatectomy, and pathologists estimated that the percentage of viable tumor cells in each sample was at least 80%. High molecular weight genomic DNA was extracted from the fresh-frozen tumor specimens and blood. Total RNA was also extracted from the tumor tissues and non-cancerous liver tissues by Trizol (Invitrogen) and their quality and quantity were evaluated by Bioanalyzer (Agilent). All subjects provided their written informed consent to participate in the study following ICGC guidelines [32]. Ethical committees at RIKEN, The Institute of Medical Science The University of Tokyo, Hiroshima University School of Medicine, Wakayama Medical University, Osaka Medical Center for Cancer and Cardiovascular Diseases, and Tokyo Women's Medical University approved this work.

### WGS analysis

Illunima library with 500-bp insert were prepared from DNAs of the tumors and lymphocytes. Sequence data was generated on the Illumina HiSeq2000 platform with paired reads of 101 bp, according to manufacturer's instruction. Mapping and identification of point mutation and somatic indels were carried out as described previously [4]. In brief, read pairs were mapped to the human reference genome using BWA [33] and possible PCR duplications were removed by samtools [34]. Point mutations and indels were identified by an in-house mutation caller, and significantly mutated genes were identified as described previously [4]. SVs were detected by the following method. Anomalous read pairs were identified and read pairs mapped within 500 bp of each other were considered to support the same rearrangement. We identified rearrangement candidates in tumor (support read pairs ≥4) and lymphocyte (support read pairs ≥1) samples, and tumor specific rearrangement candidates were selected. To exclude mapping errors, we performed a blast search of read pairs that support rearrangements against the reference genome. If a paired read was mapped with correct orientation, distance (≤500 bp) and an E-value <10^−7^ to a second location, we excluded that read pair. Reads mapped with more than two mismatches were also discarded. After filtering, candidates supported by ≥4 read pairs and at least one perfect match pair were considered as somatic rearrangements. The false positive rate was estimated to be 8.6% (15/175) by PCR verification. Since HBV genomes are polymorphic, we selected best reference HBV genomes for each sample as previously described [8]. First, we gathered unmapped reads, mapped them to 73 HBV genomes independently, and selected the HBV genome sequence with the highest number of mapped reads as the reference genome for each sample. Then, using read pairs that were mapped to both the human genome and HBV sequence, we identified integration sites supported by ≥3 read pairs [4] **.**

### RNA-Seq analysis and alignment

High-quality total RNA was subject to polyA+ selection and chemical fragmentation, and the 100–200base RNA fraction was used to construct cDNA libraries according to Illumina's protocol. RNA-Seq was performed on the HiSeq2000 platform using the standard paired-end 101 bp sequencing protocol. All sequencing reads were aligned to the known transcript sequences of UCSC known gene database (http://hgdownload.cse.ucsc.edu/goldenPath/hg19/database/knownGene.txt.gz) using Bowtie [35], with -a –best –strata -m 20–v 3 options, and the coordinates of the aligned reads were converted to the human reference genome (hg19). Unaligned reads were then aligned to the entire human reference genome (hg19) and as well as the HBV genome (AP011098) using blat [36], with -stepSize = 5 -repMatch = 2253, and aligned reads by Bowtie or blat were combined together. For each short read, the alignment with the highest number of matched bases was adopted, and the mapping quality was assigned as follows: For a location *a*, let B(a) denote the number of matched bases and let a_best_ denote the best location selected arbitrarily from those with the highest number of matched bases. The mapping quality for the read was assigned as follows:

min{100, -10×log10(1 – 1/(Σ_a_0.02^∧^(B(a_best_)-B(a))))}.

Finally, sorting and PCR duplicate removal of short reads were performed using Picard (http://picard.sourceforge.net/).

### Quantification of expression values from RNA-Seq data

To quantify expression values, we have used modified version of RKPM (reads per kb of exon per million mapped reads) measures [37]. After removing improperly aligned or low quality sequencing reads (mapping quality <60), the depth of coverage for each base in the exonic region of each RefSeq gene was tallied. Then, the numbers of bases were normalized as per kb of exon and per 100 million of aligned bases. Finally, expression value of each gene was determined by choosing the maximum of multiple RefSeq genes (if any) corresponding to the gene symbol.

### Splicing aberration detection

We detected exon skip, splice-site slip, and pseudo exon inclusion events through identification of cancer-specific spliced junctions. Spliced junctions supported by at least 4 read pairs in cancer samples less than 2 read pairs in matched liver samples, and without any annotation in RefSeq, Ensemble Gene predictions nor USCS known genes were collected. Next, the ratio of sequencing depth to the number of support read pairs for each spliced junction was compared between cancer and the matched liver samples for both sides by Fisher's exact test, and those with *P*-value <0.05 on either side were extracted. Finally, for accurately removing polymorphic splicing events, splicing junctions with the ratio between the number of supporting read pairs and the sequencing depth greater than 0.01 for at least one non-cancerous liver samples were filtered. Remained spliced junctions were categorized as follows (**Fig. 1B**): exon skip, if both junction sides were on annotated spliced junctions; splice-site slip, if one junction side was consistent with an annotated spliced junction and the other was in an intronic region; and pseudo exon inclusion, if one junction side was consistent with an annotated spliced junction and the other was in an exonic regions. Those with junctions in both sides were not consistent with annotated spliced junctions were discarded.

We detected intron retention events by evaluating the number of reads aligned on exon-intron boundaries (boundary reads), defined as those included at least 8 bp of both the exon and the intron sides of the boundaries. Among all the annotated exon-intron boundaries (RefSeq genes, Ensemble Gene predictions, and USCS known genes), we identified the candidates of intron retentions that satisfy all the following conditions: (1) The ratio between the number of boundary reads and the total reads was greater than 0.1 in the cancer sample. (2) The number of boundary reads in the cancer sample was more than 3. (3) The number of boundary reads in the non-cancerous liver sample was less than 4. (4) The ratio between the number of boundary reads and the total reads was significantly different between cancer and non-cancerous liver samples (*P*<0.05 by Fisher's exact test). (5) The ratio between the number of boundary reads and the total reads in cancer sample was significantly deviated from the beta-binomial distribution (*P*<0.0001) with parameters fitted by utilizing the number of boundary reads and the total reads for all the 22 non-cancerous liver samples at the same boundary. (6) The number of boundary reads was significantly deviated from the negative-binomial distribution (*P*<0.0001) with parameters fitted by utilizing the number of boundary reads for all the 22 non-cancerous liver samples at the same boundary.

### Detection of fusion transcripts

We developed Genomon-fusion algorism, which can detect fusion genes involving un-annotated transcripts and chimeric transcripts fused with viral sequences. Briefly, Genomon-fusion detects candidate fusion transcripts by utilizing ‘soft-clipping’ information, the unmatched parts of the partially aligned reads, along with a number of rigorously designed filters to exclude false positives often generated by ambiguous alignments to numerous repetitive or homologous sequences. First, Genomon-fusion searches for candidate fusion transcripts through genome-wide screening of the mutually nested breakpoints of soft clipped sequences. At least 2 soft clipped sequences at each breakpoint are aligned to within 10 bp of the other corresponding breakpoint, while the consistency of positions and directions of read pairs including the soft clipped sequences were investigated. Then, for each candidate fusion transcript, a set of read pairs surrounding the corresponding fusion boundary is assembled using CAP3 to constitute a contig sequence, and extract the candidate having >2 supporting read pairs that are properly aligned on that contig. Finally, for each remaining candidate, the pair of the contig split up at the fusion boundary are aligned to the human reference sequence including unplaced sequences (such as in chr1_gl000191_random and or chrUn_gl000211) by blat, and then removed the candidate if one of the two divided contigs aligned to other genomic locations with less than 3 mismatches or aligned within 1 kb of the other corresponding breakpoint.

### Detection of over-expressing genes

First, we calculated the processed expression value (PEV) for each gene, which is defined as the log_2_ of the expression values with 0.5 pseudo counts. Then, we excluded genes whose maximum PEVs among 22 cancer samples was below log_2_(1.5) or within 3 sigma from the average PEVs among 22 liver samples. Next, for each remaining gene, a Grubbs-Smirnov test for a set of PEVs among 22 cancer samples was repeatedly performed until no outliers were detected (*P*-value <0.05). The detected outliers for each gene and sample in the above procedure were identified as over-expressed genes.

### Mutation and RNA-editing detection from RNA-Seq and WGS data

Cancer-specific mutations in RNA-Seq are detected by using EBCall software [17], which can sensitively discriminate genuine mutations from sequencing errors through identification of discrepancies between allele frequencies of the candidate mutations and the distribution of sequencing errors estimated from a set of non-matched reference samples. We used the RNA-Seq data of the 22 non-cancerous liver samples as normal reference samples. We identified somatic mutations by checking the evidence in WGS data: sequencing depth ≥8 for both tumor and normal sample, allele frequencies in tumor ≥0.1, allele frequencies in normal ≤0.02, number of variant reads in tumor ≥2 and number of variant reads in normal ≤1. Furthermore, for extracting RNA editing events, we required: allele frequencies in tumor ≥0.1, allele frequencies in normal ≤0.02, and sequencing depth ≥15 for both tumor and normal samples.

### Complementary detection of GMTAs by WGS and RNA-Seq data

For rescuing point mutations or indels causing transcriptional aberrations given cancer-specific splicing aberrations detected by RNA-Seq, we searched for the variants satisfying the following. (1) The edit distance to splicing donor/acceptor motifs was changed consistent to causing the corresponding splicing aberrations. (2) The sequencing depths of tumor and normal samples were more than 9. (3) The allele frequencies of the variant were more than 10% for the tumor sample, and less than 5% for the normal sample. (4) The numbers of variant reads were no less than 3 for the tumor sample and no more than 2 for the normal sample.

For rescuing exon skips caused by SVs given SVs detected by WGS, we searched for the exon skips satisfying the following. (1) The junction points were located next or 2^nd^ next exons to the breakpoints. (2) The number of supporting reads is no less than 3. (3) The number of supporting reads for the target sample was 5 folds more than the maximum of the other samples.

For rescuing intron retentions caused by SVs detected by WGS, we searched for the intron retentions satisfying the following (1) The boundary of exon and intron was located next to the breakpoints. (2) The ratio between the number of boundary reads and the total reads was greater than 0.1 in the target cancer sample and 3 folds more than the maximum of the other samples.

## Supporting Information

S1 File **Table S1**, Clinical and pathological features of 22 HBV-associated HCCs. **Table S2**, The summary of whole genome sequencing data. **Table S3**, The summary of RNA-Seq data. **Table S4**, The summary of genome-level somatic changes identified by whole genome sequencing data. **Table S5**, List of significantly mutated genes. **Table S6**, Mutations at splicing sites and their effect on transcriptome. **Table S7**, Cancer specific splicing aberrations identified using RNA sequencing data of tumors. **Table S8**, Human-human fusion transcripts identified using RNA sequencing data of tumors. **Table S9**, Human-human fusion transcripts identified using RNA sequencing data of adjacent normal tissue. **Table S10**, HBV-human fusion transcripts identified using RNA sequencing data of tumors. **Table S11**, HBV-human fusion transcripts identified using RNA sequencing data of adjacent normal tissues. **Table S12**, Overexpressed genes with associated SVs or HBV integrations.
(XLS)

S2 File **Figure S1**, Examples of short read alignments showing splicing aberrations described with IGV (Integrative Genomics Viewer). **Figure S2**, The histogram of the number of splicing variants for each gene fusion. **Figure S3**, Spliced and un-spliced transcripts. **Figure S4**, The numbers of gene fusions detected from RNA sequencing data and those of corresponding structural variations detected in whole genome sequencing data. **Figure S5**, Ratio of FKPMs between fusion transcripts and original genes. **Figure S6**, A view of UCSC Genome Browser for gene fusions involving *NBEAP1* and non-coding RNA. **Figure S7**, Structures of several gene fusions. **Figure S8**, Histograms of breakpoint positions of inferred HBV-human fusion transcripts. **Figure S9**, Alignment status of RNA sequencing data around the *TERT* locus for RK166 cancer. **Figure S10**, HBV-*CDK15* fusion transcripts detected in RK050 cancer. **Figure S11**, RT-PCR analysis of HBV-*MLL4* fusion transcripts. **Figure S12**, The estimated expression value (FKPM) of each HBV-human fusion transcript. **Figure S13**, Evaluation of statistical significance of the number of over-expressing genes with associated structural variations or HBV integrations. **Figure S14**, Genomic and transcriptomic status of the area surrounding the *WNT1* and *WNT10B* genes in RK107. **Figure S15**, Correlation between allele frequencies of somatic mutations detected in WGS and RNA-Seq. **Figure S16**, The status of genomic and transcriptomic alterations.
(DOCX)
