# Supplementary material for: Integrated Analysis of Whole Genome and Transcriptome Sequencing Reveals Diverse Transcriptomic Aberrations Driven by Somatic Genomic Changes in Liver Cancers
Source: PLoS One. 2014 Dec 19;9(12):e114263. doi: 10.1371/journal.pone.0114263 (PMC4272259; doi:10.1371/journal.pone.0114263)
Supplement: S2 File — Figure S1, Examples of short read alignments showing splicing aberrations described with IGV (Integrative Genomics Viewer). Figure S2, The histogram of the number of splicing variants for each gene fusion. Figure S3, Spliced and un-spliced transcripts. Figure S4, The numbers of gene fusions detected from RNA sequencing data and those of corresponding structural variations detected in whole genome sequencing data. Figure S5, Ratio of FKPMs between fusion transcripts and original genes. Figure S6, A view of UCSC Genome Browser for gene fusions involving NBEAP1 and non-coding RNA. Figure S7, Structures of several gene fusions. Figure S8, Histograms of breakpoint positions of inferred HBV-human fusion transcripts. Figure S9, Alignment status of RNA sequencing data around the TERT locus for RK166 cancer. Figure S10, HBV-CDK15 fusion transcripts detected in RK050 cancer. Figure S11, RT-PCR analysis of HBV-MLL4 fusion transcripts. Figure S12, The estimated expression value (FKPM) of each HBV-human fusion transcript. Figure S13, Evaluation of statistical significance of the number of over-expressing genes with associated structural variations or HBV integrations. Figure S14, Genomic and transcriptomic status of the area surrounding the WNT1 and WNT10B genes in RK107. Figure S15, Correlation between allele frequencies of somatic mutations detected in WGS and RNA-Seq. Figure S16, The status of genomic and transcriptomic alterations. (DOCX) [file pone.0114263.s002.docx]

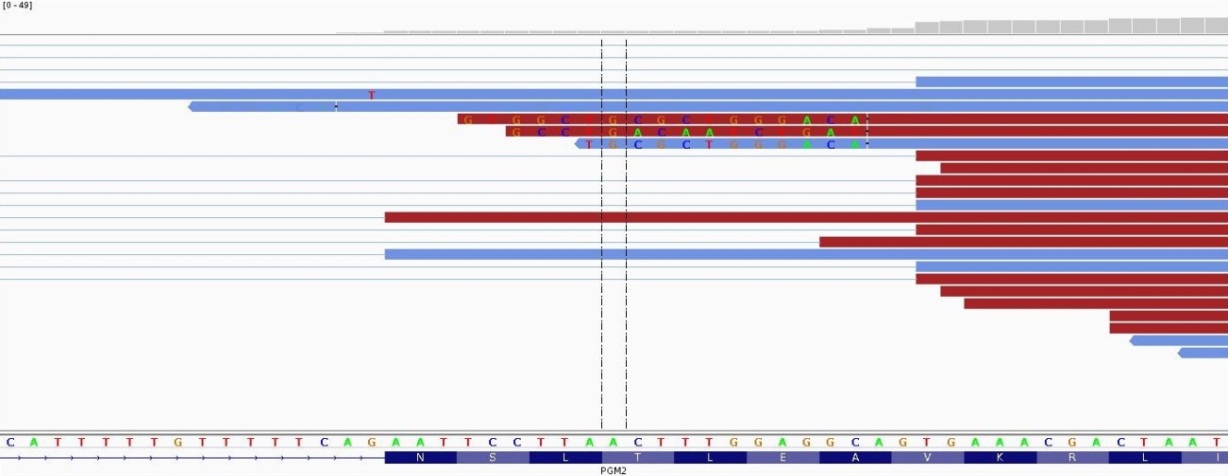


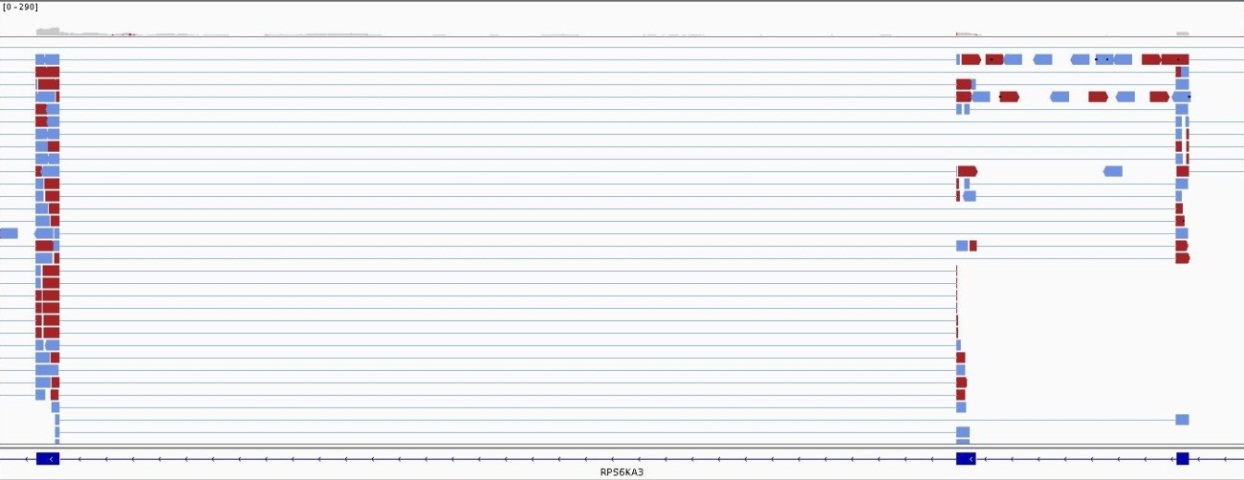


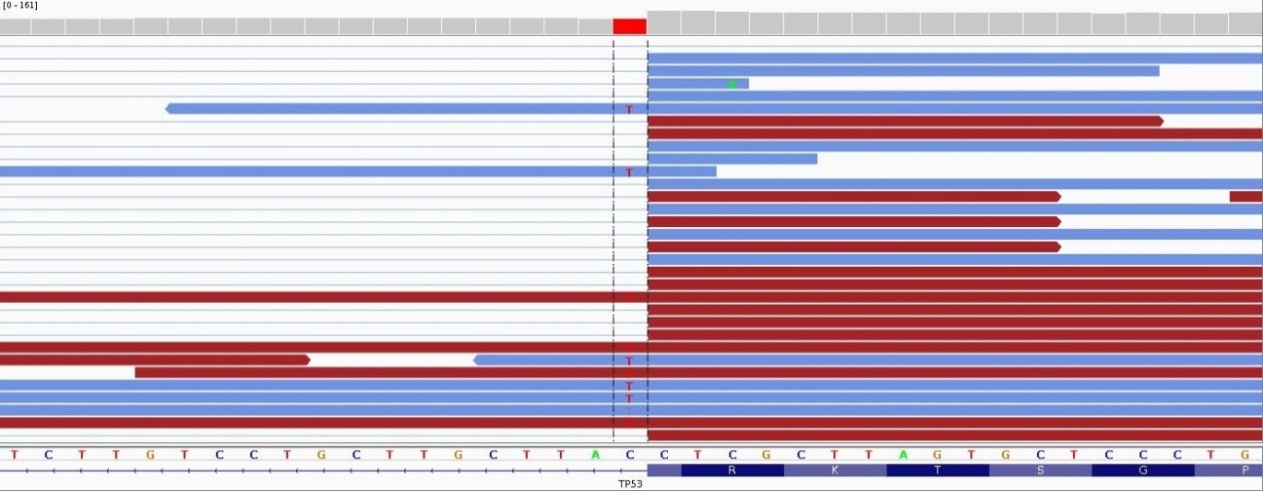


**Figure S1**: Examples of short read alignments showing splicing aberrations described with IGV (Integrative Genomics Viewer). (Upper) By a substitution at the splicing acceptor site (AG->AT), splicing sites are forwarded to the next or further next AG sites. (Middle) An exon skipping is caused by a mutation of a splicing acceptor site. (Lower) An intron retention driven by a substitution at the splicing donor site (C->T). The expression of the substituted allele (T) is observed specifically within the set of un-spliced short reads.


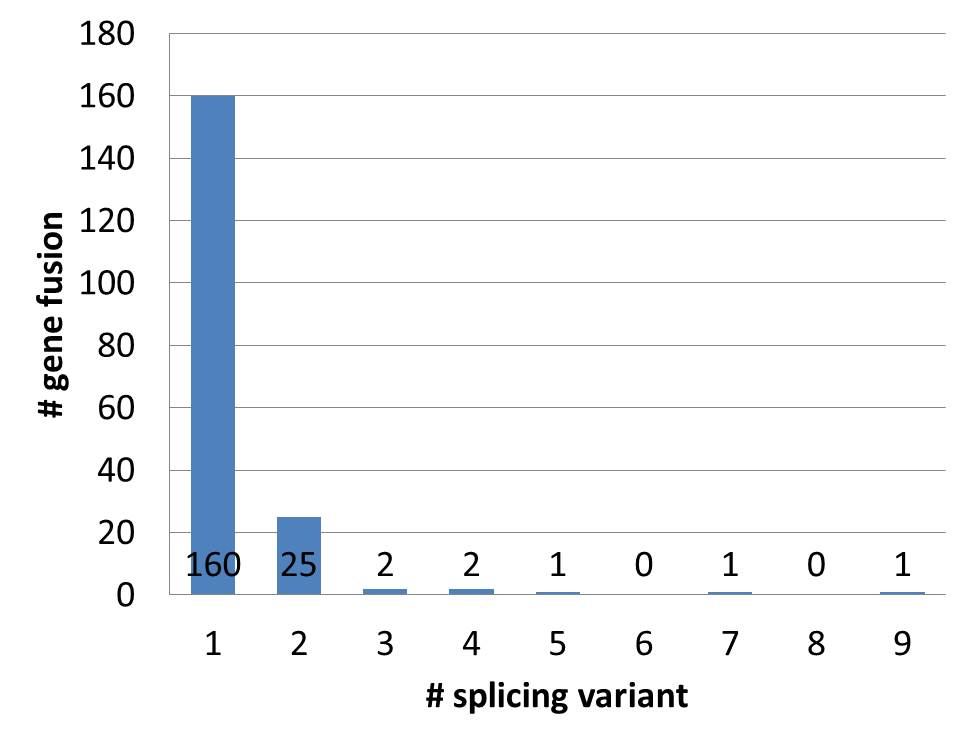


**Figure S2**: The histogram of the number of splicing variants for each gene fusion.

(A)


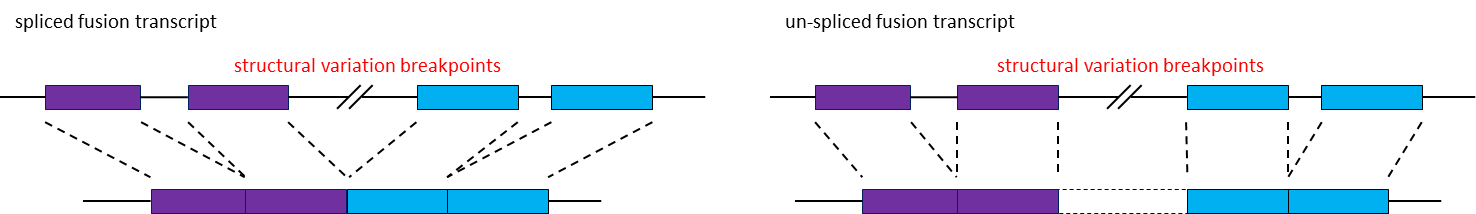


(B)


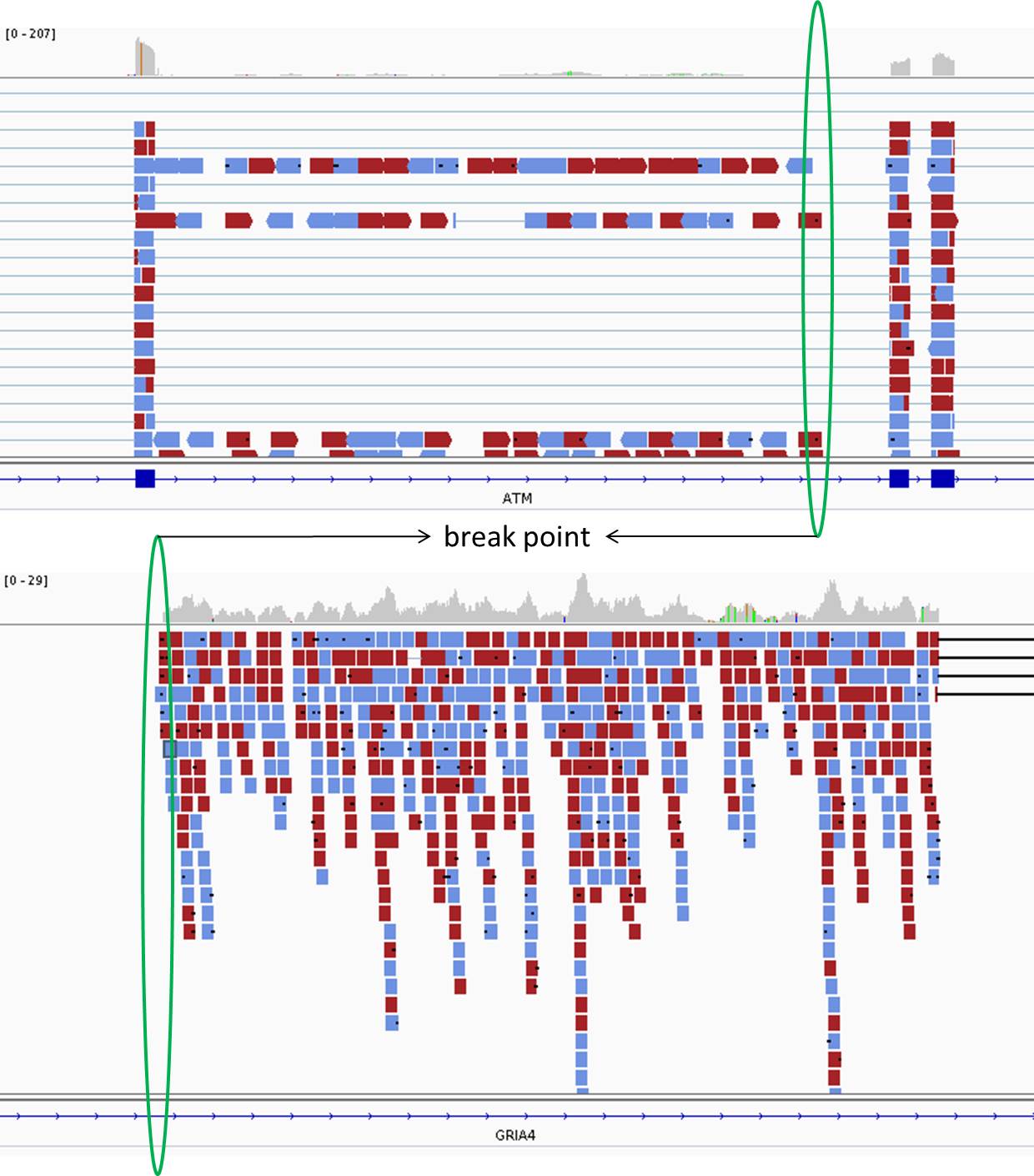
**Figure S3**: Spliced and un-spliced transcripts. (A) In a typical mechanism for the formation of fusion transcripts, hybrid gene transcripts derived from somatic structural variations with breakpoints located mostly in intronic regions are processed by splicing. On the other hand, un-spliced fusion transcripts share the breakpoints with their corresponding genomic structural variations. (B) Alignment status of RNA-Seq data around breakpoints of *ATM*-*GRIA4* structural variation. Although the directions of the breakpoints are consistent with the directions of transcription for both genes, there were no spliced fusion transcripts observed.


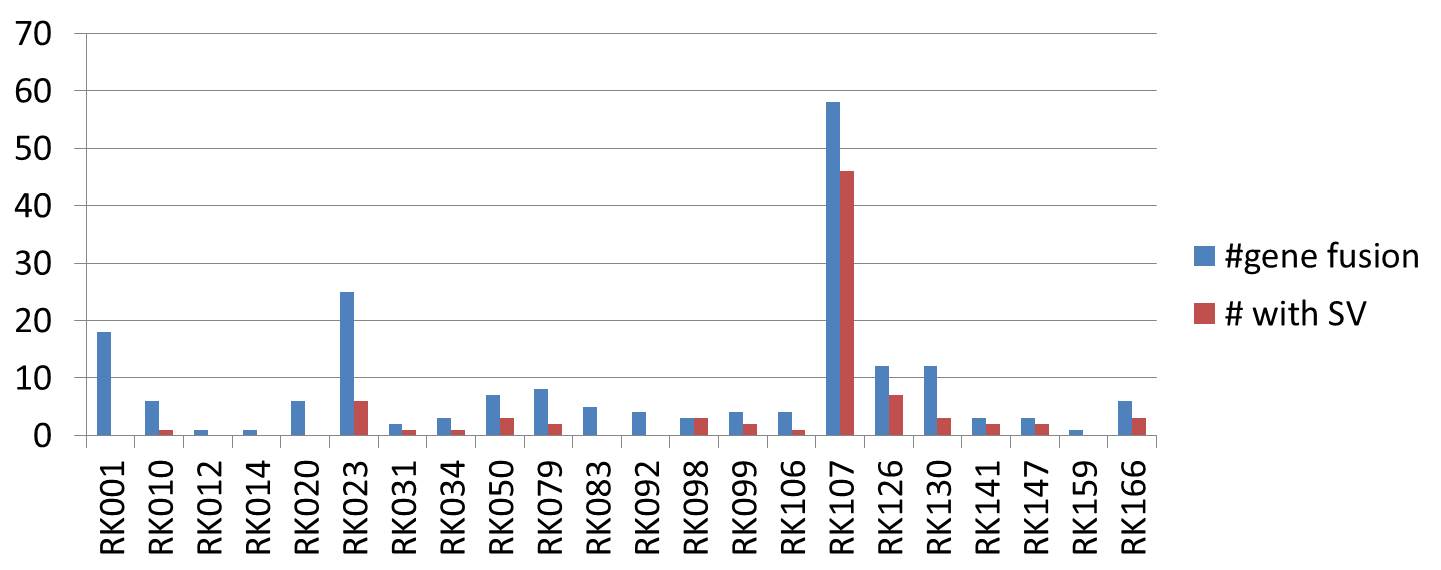


**Figure S4**: The numbers of gene fusions detected from RNA-Seq data and those of corresponding structural variations detected in whole genome sequencing data.


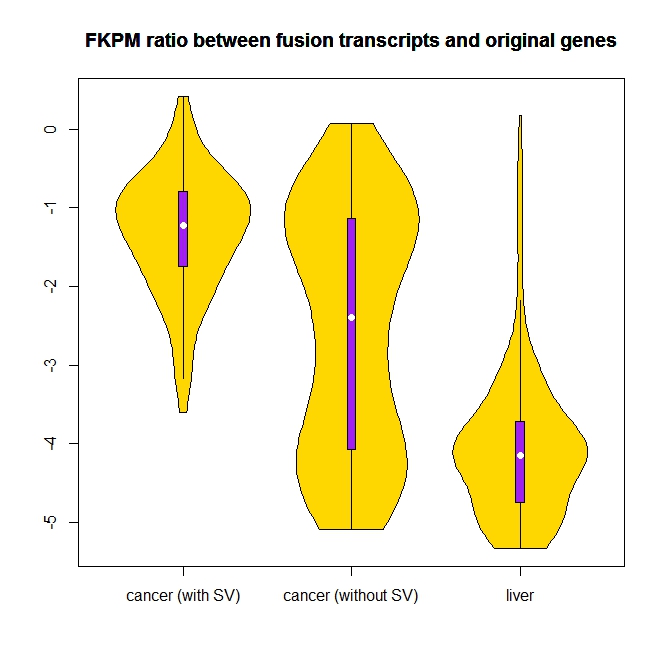


**Figure S5**: Ratio of FKPMs between fusion transcripts and original genes (the genes with higher expression values were adopted for ratio calculations) for (1) fusion transcripts detected in cancer samples having associated structural variations, (2) those detected in cancer samples without associated structural variations, and (3) those detected in non-cancerous liver samples. Fusion transcripts with associated structural variations tend to have higher ratios. The ratios in those without associated structural variations shows a bimodal distribution. The cluster with higher ratios may be caused by false negative detections of structural variations in whole genome sequencing data, while the clusters with lower ratios may be derived from fusion transcripts in minor sub-populations.


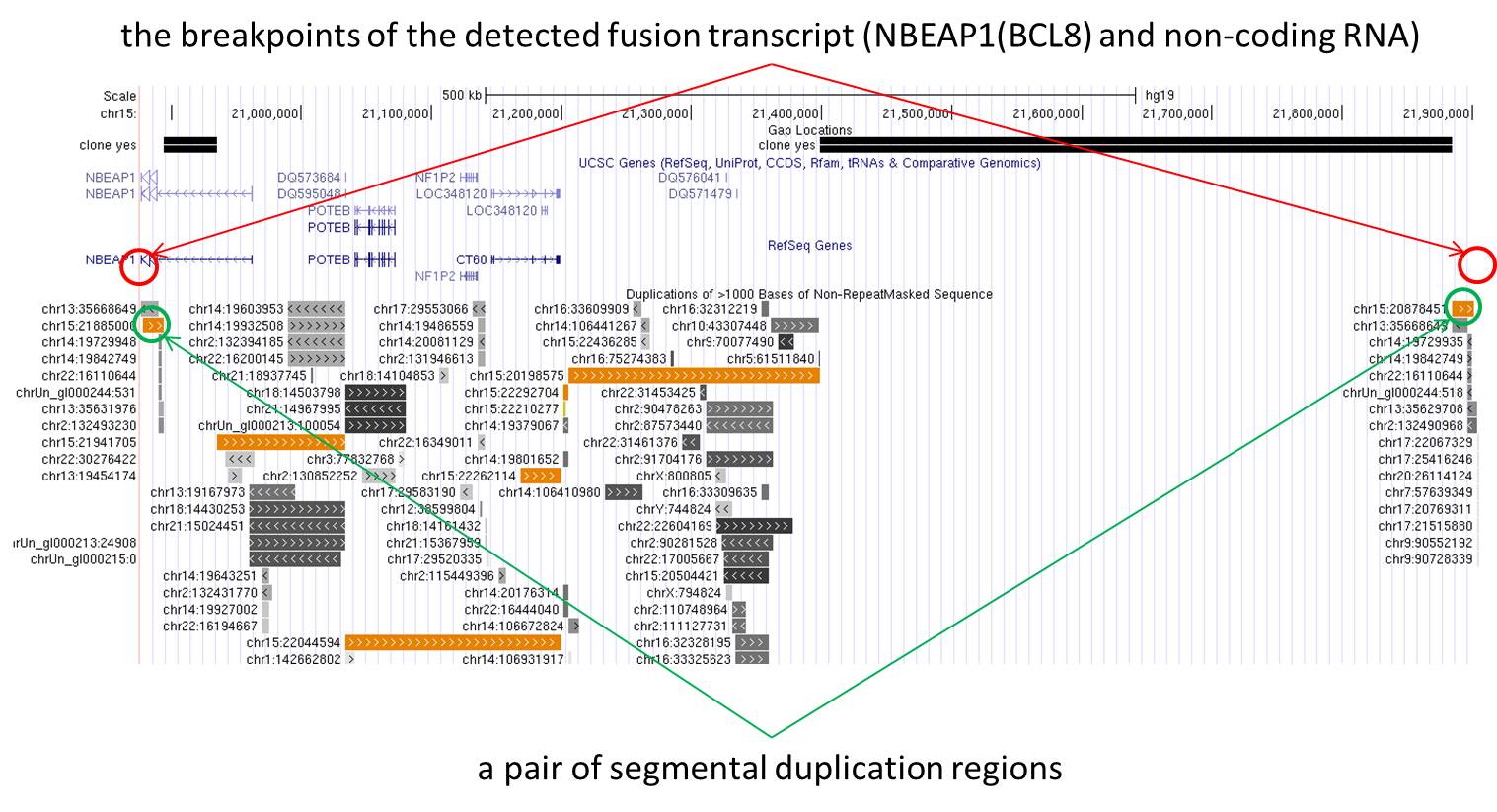


**Figure S6**: A view of UCSC Genome Browser (http://genome.ucsc.edu/) for gene fusions involving *NBEAP1* and non-coding RNA. Red circles represent the breakpoints of the detected fusion transcripts. Green circles represent a pair of segmental duplication regions, which are near-identical (>= 99%). Although we could find a few read pairs supporting the rearrangement corresponding to this gene fusion in WGS data, we could not exclude the possibility that these supporting reads were due to alignment artifacts because of high similarity between the regions around the two breakpoints.


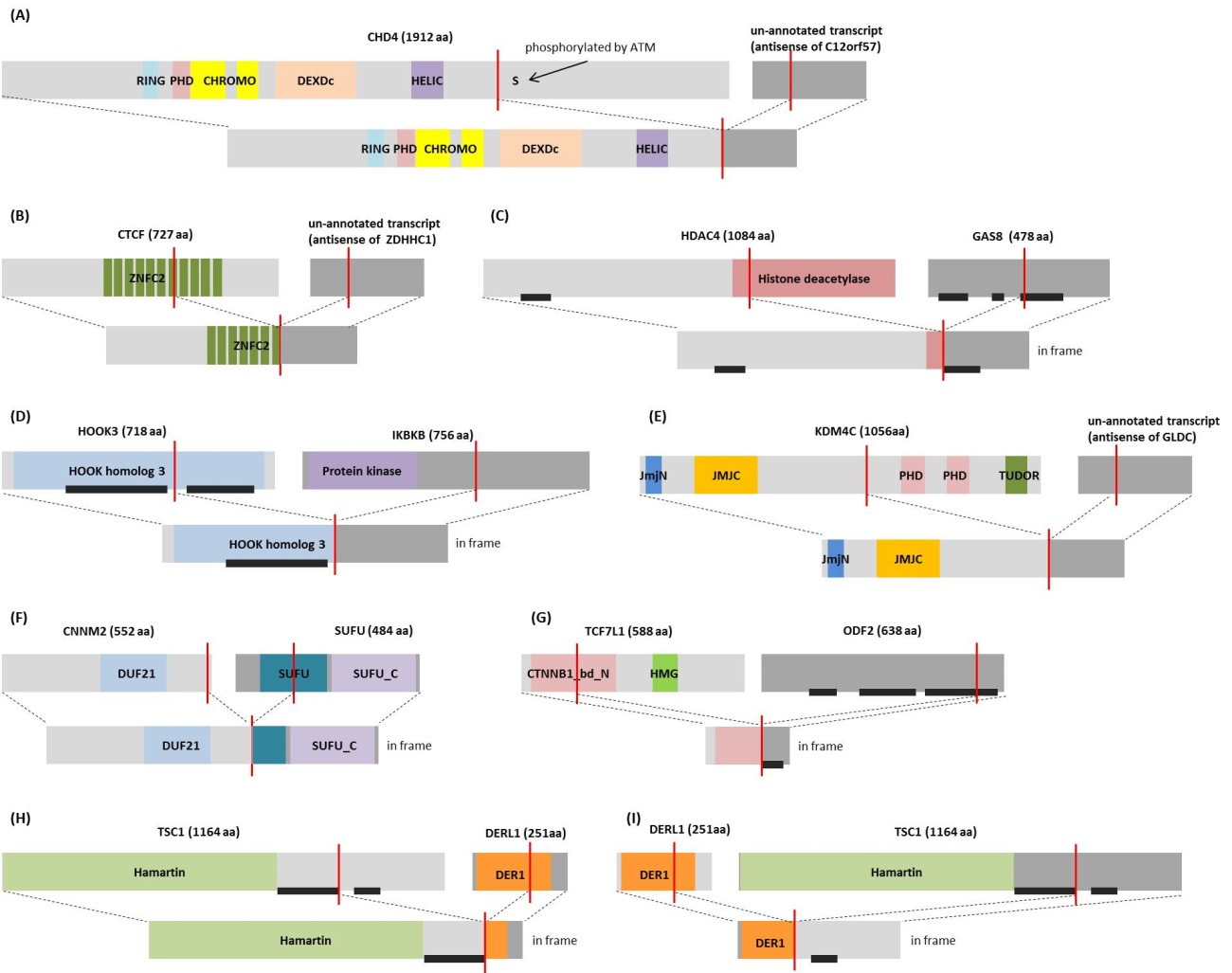


**Figure S7**: Structures of several gene fusions. Red lines represent breakpoints leading gene fusions. HDAC4, GAS8, HOOK3, ODF2 and TSC1 have coiled coil motifs represented by black rectangles below the gene bars. Resultant fusion transcripts lack (A) the reportedly phosphorylation site targeted by ATM, (B) zinc finger domains, (C) histone deacetylase domain, (D) protein kinase domain (E) two PHD zinc finger domains and tudor domain, (F) SUFU domain (G) HMG domain (H) coiled coil motifs, and (I) hamartin domain.


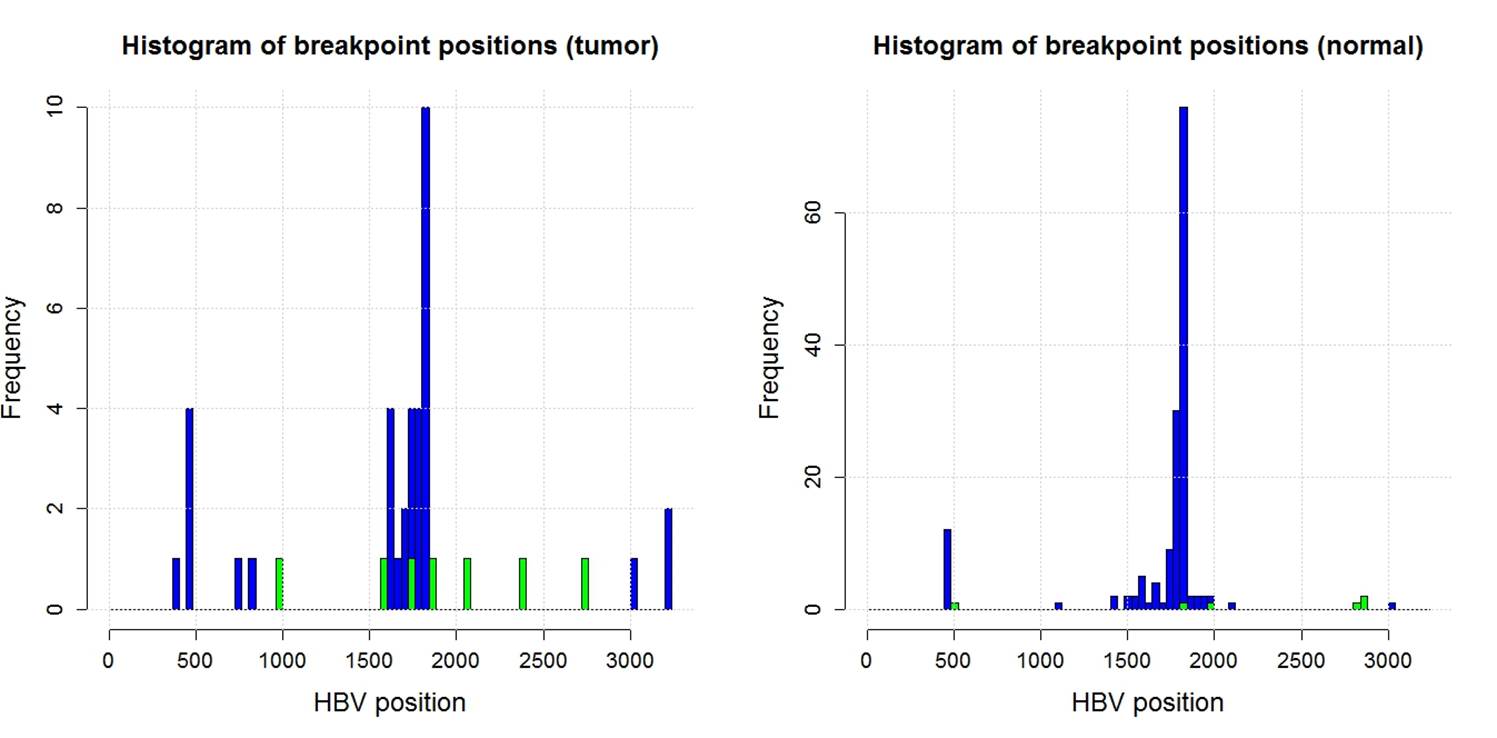


**Figure S8**: Histograms of breakpoint positions of inferred HBV-human fusion transcripts. Blue and green bars show the frequencies of breakpoints directing to the plus and minus strands, respectively. Compared to breakpoint positions detected in tumors, those detected in adjacent non-cancerous tissues are more concentrated on the locus 1770 ~ 1830 where the HBx genes are located or on the HBV fusion splicing hotspot (458bp).


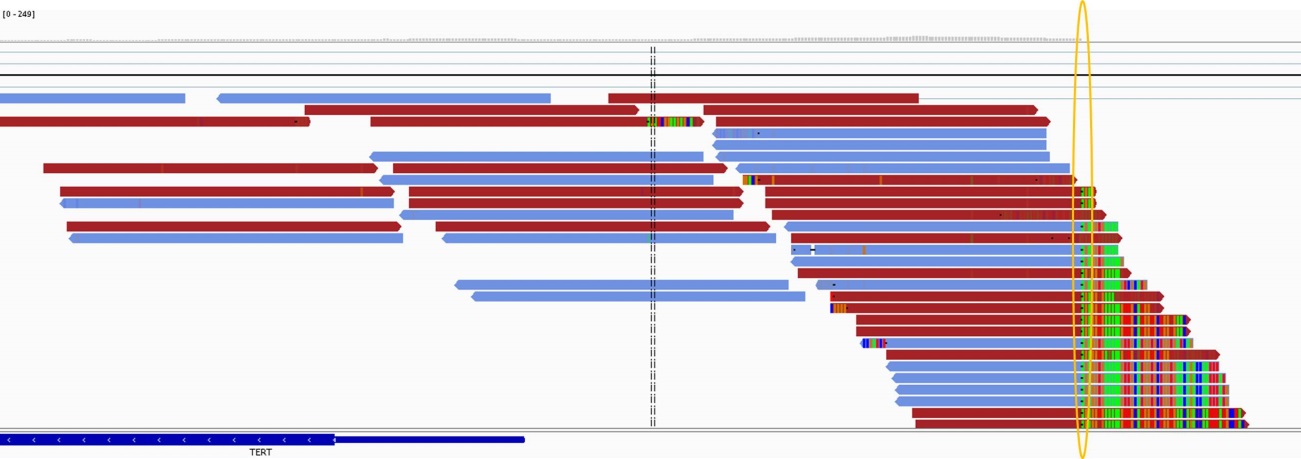


**Figure S9**: Alignment status of RNA sequencing data around the *TERT* locus for RK166 cancer. A HBV integration occurs just upstream of the transcription start site of *TERT* (surrounded by orange circle), where parts of short reads, which are actually derived from HBV sequences, are soft clipped and marked by different colors. Transcripts generated from the HBV-integrated locus are directly concatenated to the transcription start site of the *TERT* gene, leading to over-expression of the full-length transcript.


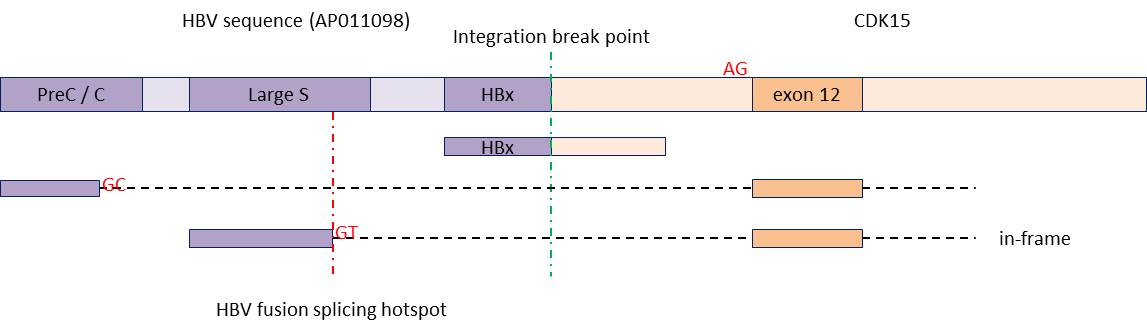


**Figure S10**: HBV-*CDK15* fusion transcripts detected in RK050 cancer. One transcript was an un-spliced transcript having the same breakpoint as the genomic integration breakpoint, and the others existed in spliced forms. One transcript spliced at the HBV fusion splicing hotspot and was inferred to be in-frame.


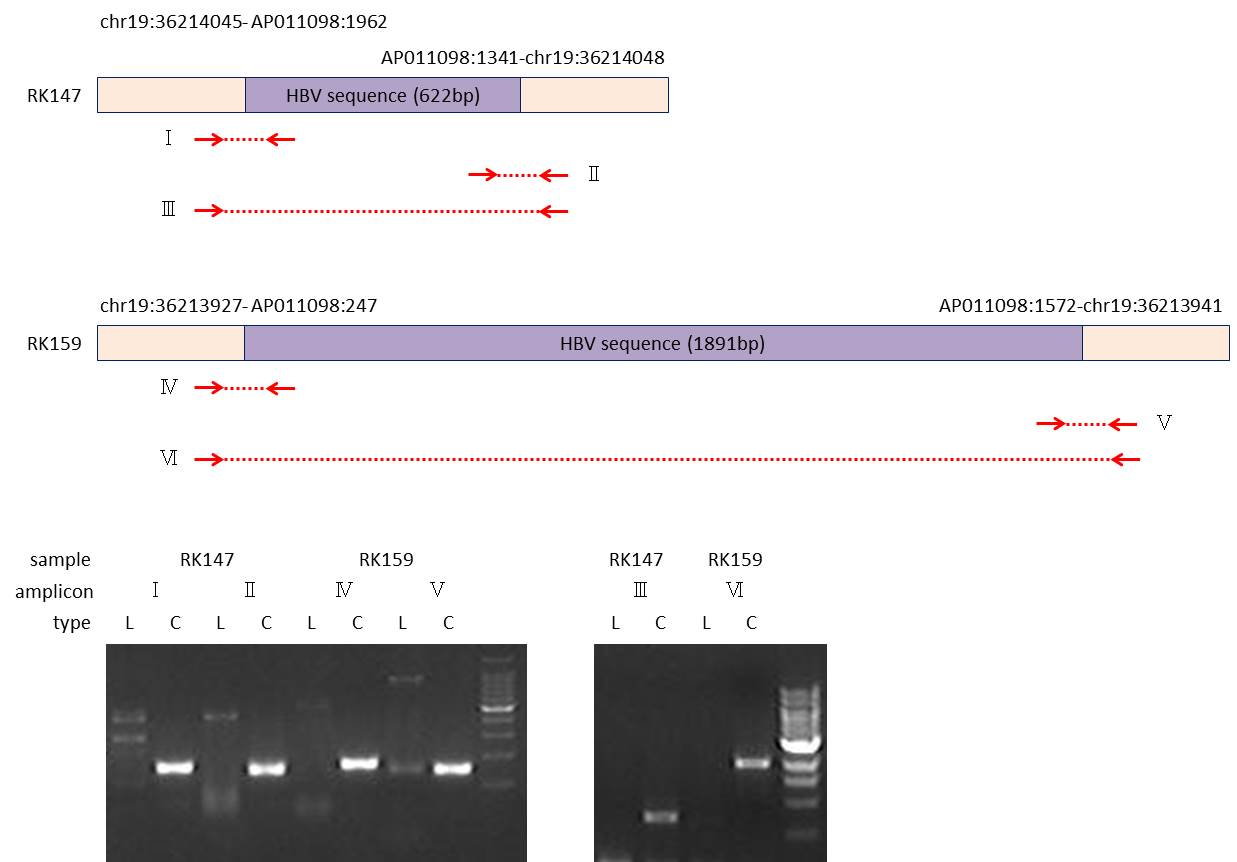


**Figure S11**: RT-PCR analysis of HBV-*MLL4* fusion transcripts. Primers were designed for HBV-*MLL4* fusion transcripts detected by RNA-seq (I, II, IV, V), each of which involves the sequence around the breakpoint of HBV integration. and presumed full *MLL4*-HBV-*MLL4* transcripts (III, VI) containing all the integrated HBV sequences.


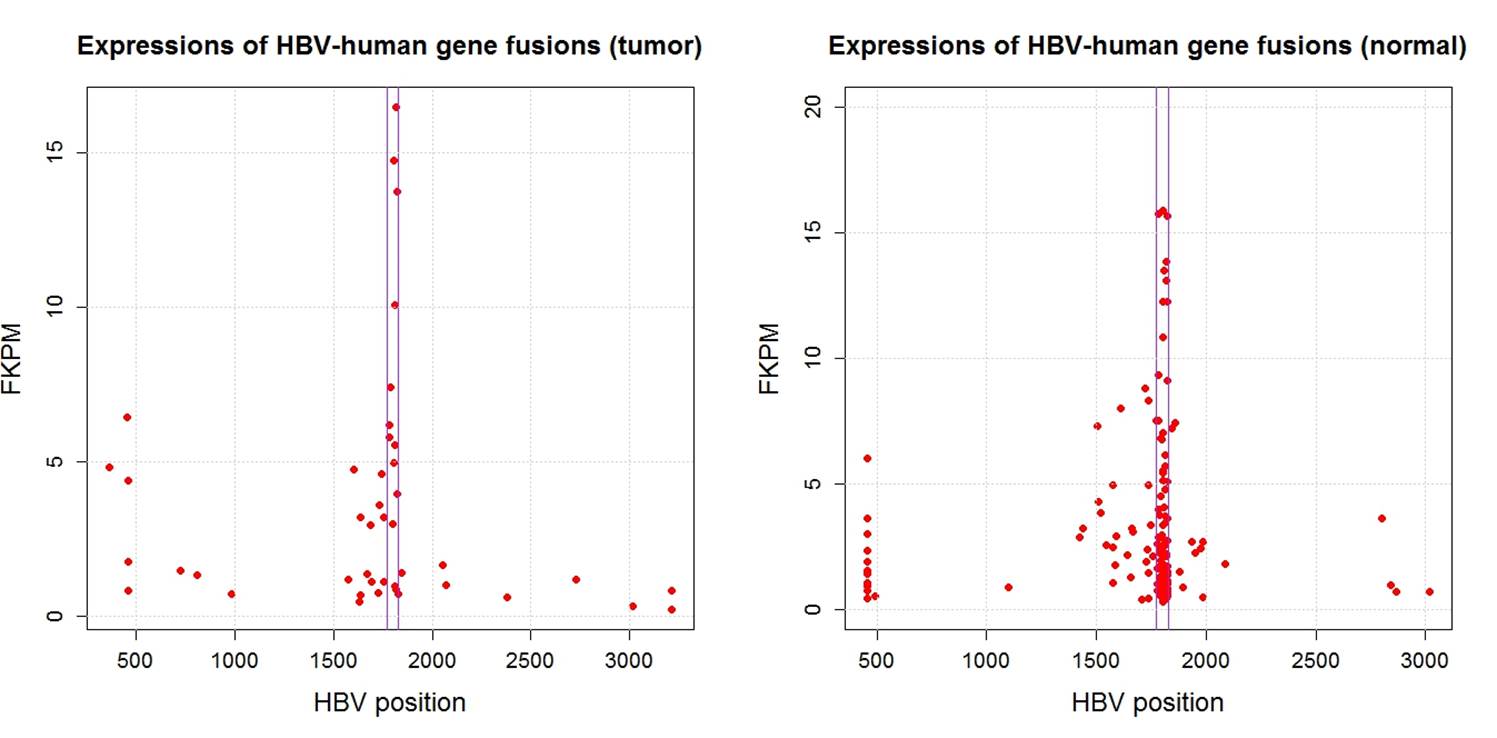


**Figure S12**: The estimated expression value (FKPM) of each HBV-human fusion transcript (red circle) depending on the coordinates of fusion breakpoints. Expression levels of transcripts within the locus where breakpoints were concentrated (1770 – 1830, surrounded by purple vertical lines) were statistically significantly higher than those in other regions (*P* = 5.01×10^5^, Wilcoxon rank sum test).


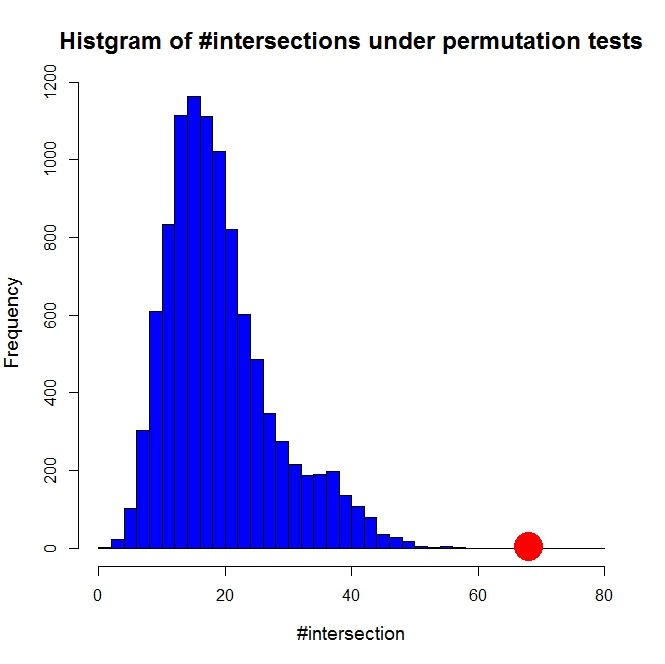


**Figure S13**: Statistical significance of the number of over-expressing genes with associated structural variations or HBV integrations (red circle) was evaluated against the 10,000 permutated combinations under the null hypothesis (histogram in blue).


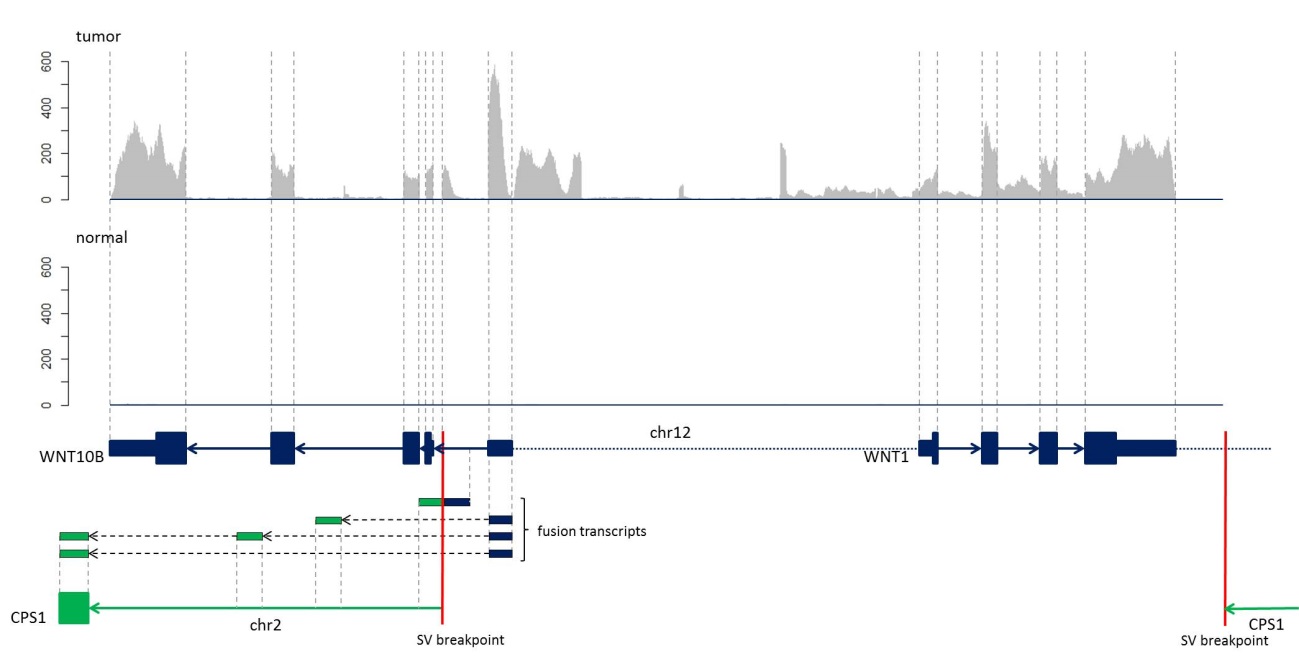


**Figure S14**: Genomic and transcriptomic status of the area surrounding the *WNT1* and *WNT10B* genes in RK107. The two plots above show the normalized (per 100M aligned reads) count of mapped bases in the RNA sequence data for tumor and normal samples. Below, boxes, real lines and dotted line show exonic, intronic and intergenic regions, respectively. Two translocations with *CPS1* loci were detected by whole genome sequencing data (shown by red vertical lines). At the site of one translocation breakpoint, we detected four discrete WNT10B-fusion transcripts; one fusion transcript shared the same breakpoints with the corresponding genomic translocation (un-spliced fusion transcript), and the other were in spliced forms.

**Figure S15**: To investigate the relationship of allele frequencies of somatic mutations in WGS and RNA-Seq, we filtered the somatic mutations with lower sequencing depths either in WGS or RNA-Seq (<=50) for accurately obtaining variant allele frequencies. Also, we just focused on somatic substitutions because accurate estimation of allele frequencies for indels is difficult due to redundancy of short read alignment especially in RNA-Seq where we have to treat splicing variant problems. Yet, 263 somatic substitutions were remained for investigation. There is a certain level correlation between the allele frequencies of somatic mutations found to be highly confident in WGS and RNA-Seq (correlation efficiency =0.466, *P-*value =2.2 x10^-16^ by Pearson's product-moment correlation).

(A)


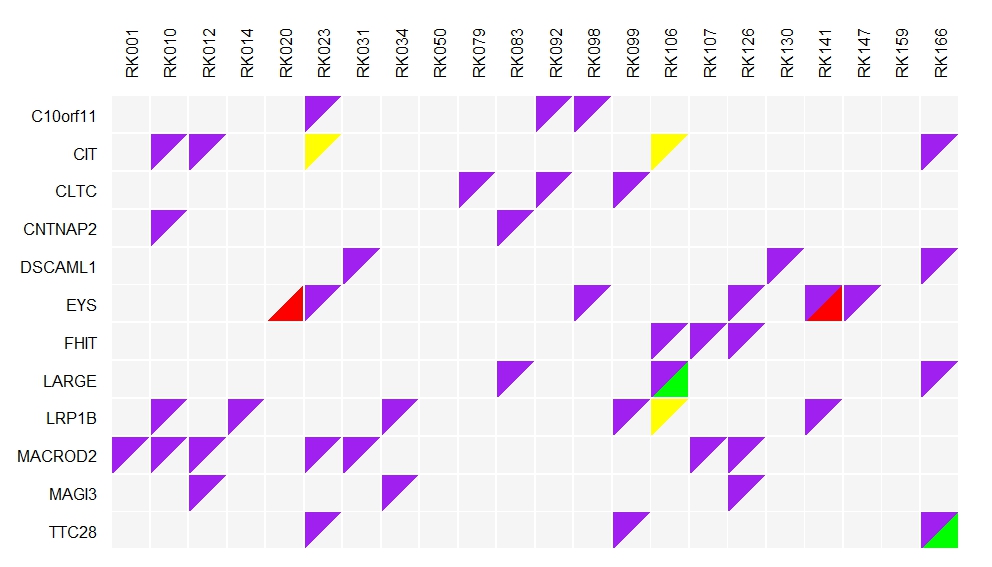


(B)


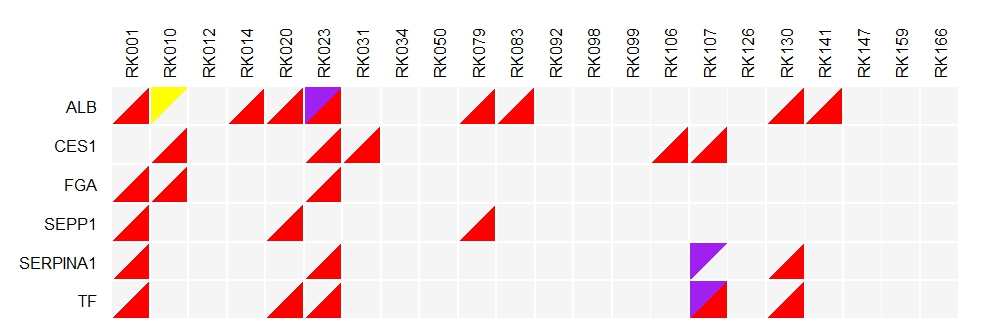


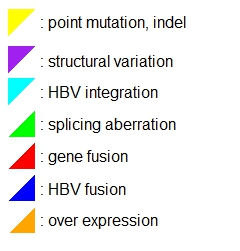


**Figure S16**: The status of genomic and transcriptomic alterations of genes with (A) recurrent structural variations (>= 3 HCCs), and (B) recurrent gene fusions (>=3 HCCs).
